# A smart energy management system for surface unmanned vehicles for border surveillance missions

**DOI:** 10.1038/s41598-025-08579-x

**Published:** 2025-07-03

**Authors:** João F. P. Fernandes, Mário Assunção, Daniel Serrano, Pedro Afonso, Pedro Pinheiro, Hugo Marques, José Neves, Pedro Teodoro, Ricardo Póvoa, Rosa Marat-Mendes, P. J. Costa Branco

**Affiliations:** 1 IDMEC, Instituto Superior Técnico, Universidade de Lisboa, Lisboa, Portugal; 2 Escola Superior Náutica Infante D. Henrique, Paço de Arcos, Portugal; 3 Instituto Superior Técnico, Universidade de Lisboa, Lisboa, Portugal; 4 Instituto de Telecomunicações, Lisbon, Portugal

**Keywords:** Unmanned surface vehicle, Modelling, Energy management system, Optimization, *A-Star*, * Bidirectional graph-based*, Electrical and electronic engineering, Energy storage, Renewable energy

## Abstract

This work proposes a methodology to extend the range of marine unmanned surface vehicles (USV) for border surveillance missions. The typical small scale of USVs and their lack of in-board pilots make USVs an important tool for remote applications, such as border surveillance missions and for dangerous areas operations. However, also due to their small scale, their mission range is typically limited. In this paper, it is proposed a combination of a smart energy management system (SEMS) with electric propulsion and photovoltaic panels to find the optimal path and speed capable of extending the mission range. The developed SEMS is capable of planning a mission profile based on the predicted environmental conditions. To achieve this, one presents a new A-star algorithm with probabilistic behaviour to avoid local minimums and find alternative paths that would reduce the energy consumption in later hours of the mission. The developed system was included in a USV prototype and tested under real environmental conditions at the interface between the Tejo River and the Atlantic Sea, in Lisbon, Portugal. Experimental results showed that the inclusion of photovoltaic panels and the SEMS allowed for planning the mission including the time-variable environmental conditions, leading to an extension of up to 50% of the mission range.

**Supplementary Information:**

The online version contains supplementary material available at 10.1038/s41598-025-08579-x.

## Introduction

Unmanned marine surface vehicles (USVs) present many advantages for marine missions and applications, thus studies and developments that aim both efficient power generation and management as well as total autonomous navigation, particularly focused on ship collision and object avoidance, have been a hot topic over the last years^1–6^. In fact, these marine crafts typically present higher efficiencies and energy-saving qualities, while presenting higher safety due to their lack of pilot in-board. The types of USVs vary from being remotely operated to being partially or fully autonomous^7–9^. These can be used as generic platforms equipped with different sensors and actuators to match different applications and even adapted from existing manned vehicles^10^.

Due to their remote operation, the USVs are suitable for various applications. For example, in dangerous areas, such as polluted areas, harsh environments, or nuclear-contaminated sites, these may offer significant advantages to carry out different missions^11^. USVs generally have reduced autonomy, payload and power and are small-scale, scientific research is mainly responsible for the rapid growth in USV production^12^. USVs are also being explored to execute military and defence missions, such as border surveillance, and perimeter defensive confrontation^13^. Due to their remote operation and lower costs, multiple USVs can be used to form a surveillance grid, operated remotely, to perform multiple tasks at a large scale^14^.

Therefore, the use of USV is an important research topic related to the objectives of FRONTEX^15^ in the development of technologies for Border Surveillance and Situational Awareness. One possible solution to extend the limited range of USVs is to include photovoltaic panels (PV)^16^ and use energy management systems to plan an optimized mission.

There are some successful projects employing solar energy in their marine surface vehicles. The Osaka Prefecture University proposed a solar-powered 2.87 m long autonomous surface vehicle (ASV) with 200 W of photovoltaic panels^17^, the Emergent Space Technologies, Inc. proposed a 5.5 m long ASV with PV panels for oceanographic and atmospheric research^18^, and the Villanova University proposed a hybrid USV with PV panels and fuel cells^19^. Using solar energy as a complementary energy source has shown to be viable for these applications, however, it presents some drawbacks that must be addressed^20^. The first is related to the limited power provided by the solar panels, and their unpredictable disturbances due to weather changes, which typically do not cover the required energy for propelling the vehicle. The second is related to the influence of sea salinity on the degradation of PV systems, requiring additional maintenance to keep adequate levels of performance. Therefore, the use of solar energy should be seen as a complementary source, combined with other energy systems and proper maintenance.

When dealing with limited energy and multiple energy sources, it becomes important the use of Energy Management System (EMS) for optimal operability, for example, to optimize fuel consumption and mission range or emissions^21–25^. In^26^, the authors proposed a non-linear model predictive control for optimizing the speed reference and use of combustion engine and electric motors, for a hybrid ship propulsion, by developing a model of the ship for typical load profiles, using clustering and machine learning. Another important method is related to finding the optimal mission path, that invites the usage of computational intelligence in the decision-making process prior to and during navigation^27^. This is relevant for large patrol missions where different weather and sea conditions can occur. One way is to discretise the patrol map into small sections and use optimization techniques to find the best path^28–33^, . However, this is not a trivial problem when environmental conditions are changing throughout the mission, as reported in^34^, which can have an impact on stability if dynamics are unknown or not accurate^35^.

Following the previous strategies, the SEMS4USV project, financed by FRONTEX Research Grants^15^, deals with the need to develop advanced technologies for environmentally sustainable systems and operations in border surveillance missions. Its objectives are: (a) to demonstrate the feasibility of a fossil-free unmanned marine surface vehicle with electric propulsion and photovoltaic panels, to extend the time and range of border surveillance missions; (b) to develop a smart energy management system (SEMS), considering the prediction of weather and sea conditions and the mission profile, for the mission planning, to maximize the mission time and range, while minimizing risks; and (c) to demonstrate the integrated USV SEMS prototypes in real river/sea environments.

**Fig. 1:**
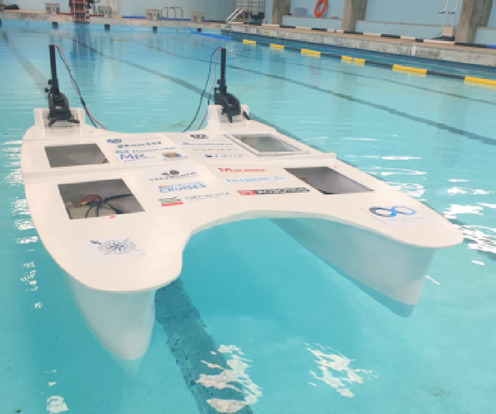
Unmanned surface vehicle initial prototype.

To support these objectives, this paper proposes a novel methodology to extend the range of marine USV for border surveillance missions. The novelty resides in the development of a SEMS capable of handling time-varying environmental conditions, photovoltaic panels and batteries, and ensure robust implementation strategy under real environmental conditions. The SEMS was developed to plan the mission actions that optimize the USV’s operational time, considering predicted weather and rives/sea conditions and mission requirements. The proposed methodology was verified and implemented in the USV-enautica1 shown in Fig. 1.

“Research methodology” of this paper outlines the proposed research methodology. “USV prototype and modelling” details the USV prototypes and its modelling while “USV model validation” validates the prototype through experimental tests under real environmental conditions. In “Smart energy management system”, the Smart Energy Management System is proposed and compared against other techniques, and it is validated through simulations. Finally, “Experimental results” presents the experimental validation of the complete system under real environmental conditions at the interface between Tejo River and the Atlantic Sea in Lisbon, Portugal.

## Research methodology

**Fig. 2:**
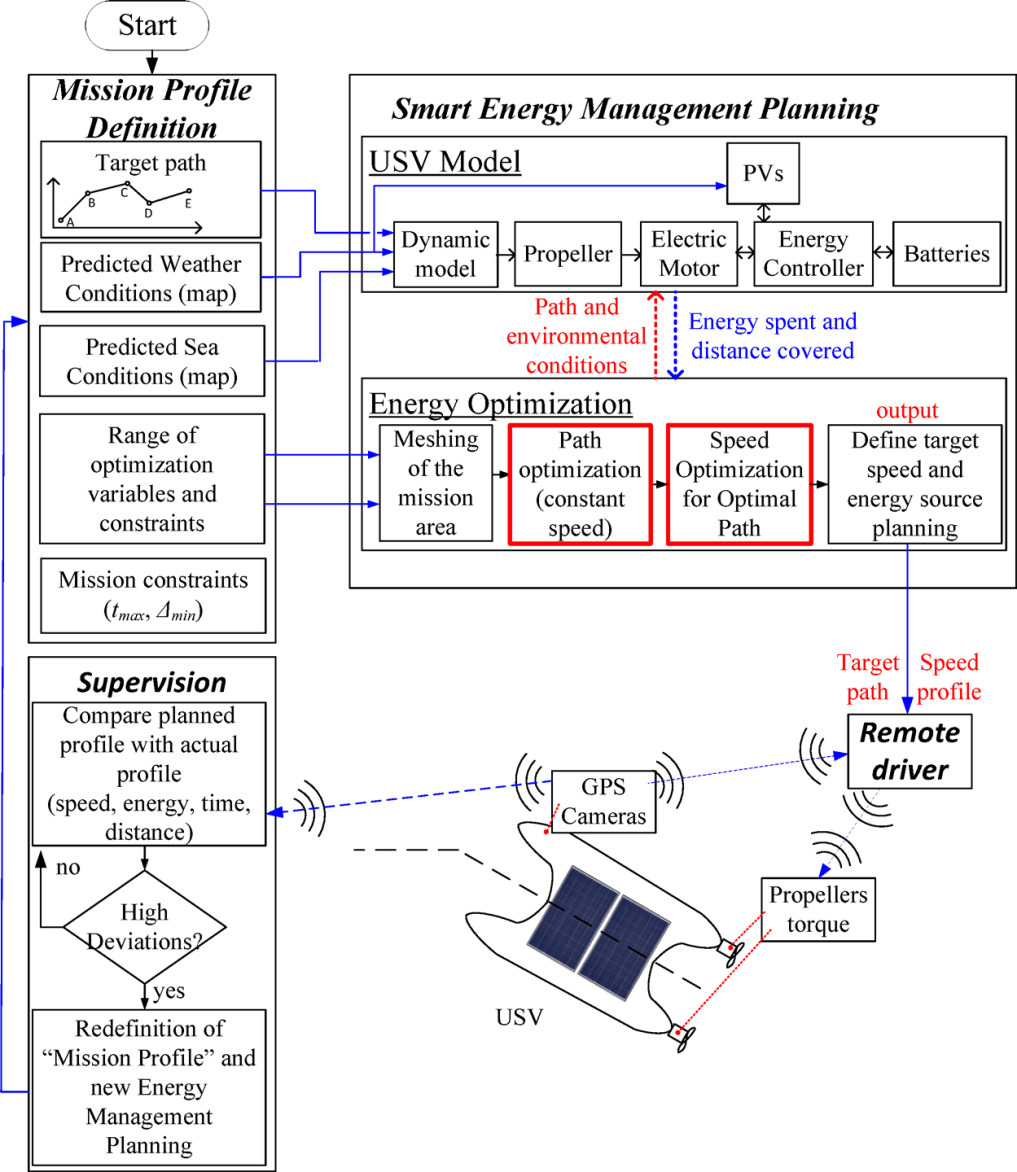
Proposed solution for the USV system with extended autonomy.

The proposed solution for extending the range of the USV is shown in Fig. 2. The methodology starts by defining the mission profiles and targets for the border surveillance mission and by gathering data for the mission’s environmental conditions. Then, this information is provided to the Smart Energy Management System (SEMS), which is formed by a model of the USV and the energy optimization system. The SEMS optimizes the USV path and speed to extend the USV mission range and provides the remote driver with an optimized energy consumption mission profile. The remote driver, then, controls the USV following the optimized profiles, and a supervision system monitors the mission. If high deviations are found, the supervision system enquires the SEMS for a new mission recalculation based on new updated environmental conditions or mission targets.

In^36^, the authors implemented a bidirectional graph to plan the mission, however, it was concluded that it was only suitable for short missions due to the constant change in environmental conditions. Therefore, it is now extended the previous solution by developing a new A-star-based approach with probabilistic behaviour to handle the time-varying environmental conditions, which could not be considered using graph-based algorithms. The proposed SEMS follows a three-steps methodology: (1) Meshing of the mission area, (2) Path optimization at a constant speed and (3) Speed optimization for the optimal path.

The robustness of the whole system was verified under real environmental conditions at the interface between Tejo River and the Atlantic Sea in Lisbon, Portugal. The experimental validation of the developed extended-range USV prototype with electric propulsion, photovoltaic systems and with the SEMS system was carried out under river/sea conditions, following a typical profile of a border surveillance mission. The performance (range, mission time and manoeuvrability) of the developed USV prototype was compared with the initial prototype, USV-enautica1.

## USV prototype and modelling

This section describes the developed USV prototype, followed by an overview of its modelling process.

### USV prototype

**Fig. 3:**
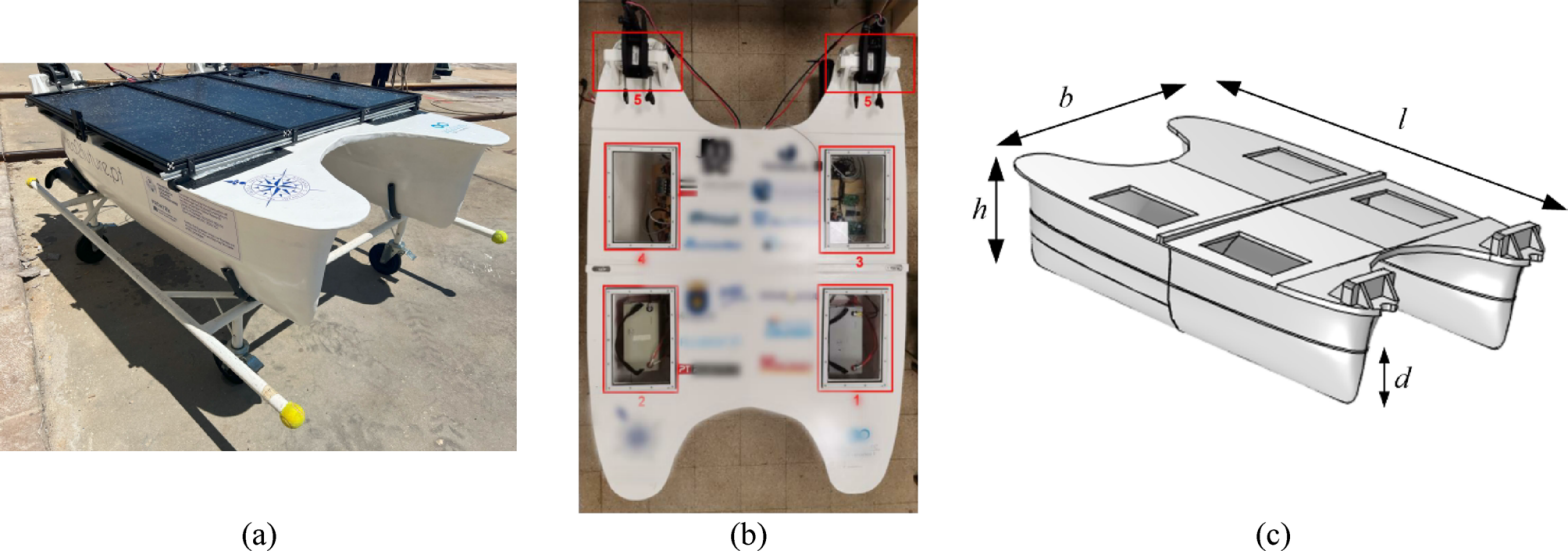
The USV in study. (**a**) Overview, (**b**) top view, (**c**) 3D model with their dimensions of Table 1.

The USV in this study is a low-weight composite catamaran vessel, displayed in Fig. 3. Its main materials are glass fibre reinforced polymer (GFRP) in the hull and plywood core reinforced with GFRP faces for the sandwich top cover. The USV main dimensions are presented in Table 1. The hull contains four compartments with sandwich shelves made of FGRP faces with foam core, where most of the components of the USV are located, Fig. 3b “Introduction”, “Research methodology”, “USV prototype and modelling” and “USV model validation”. Each of them is covered by one acrylic hatch, providing easy access to the components inside them, while keeping the hull watertight. At the rear of the USV where the propulsors are tied (“Smart energy management system”), there are wood structures with GFRP faces. The solar panels are placed on top of an aluminium structure that allows them to be lifted to access the hatches.

**Table 1:** USV mechanic parameters.

Parameter	Value
Length, *l*	215.5 cm
Beam, *b*	137.2 cm
Height, *h*	45.0 cm
Draft, *d*	24.9 cm
Weight, *w*	30 kg

**Fig. 4:**
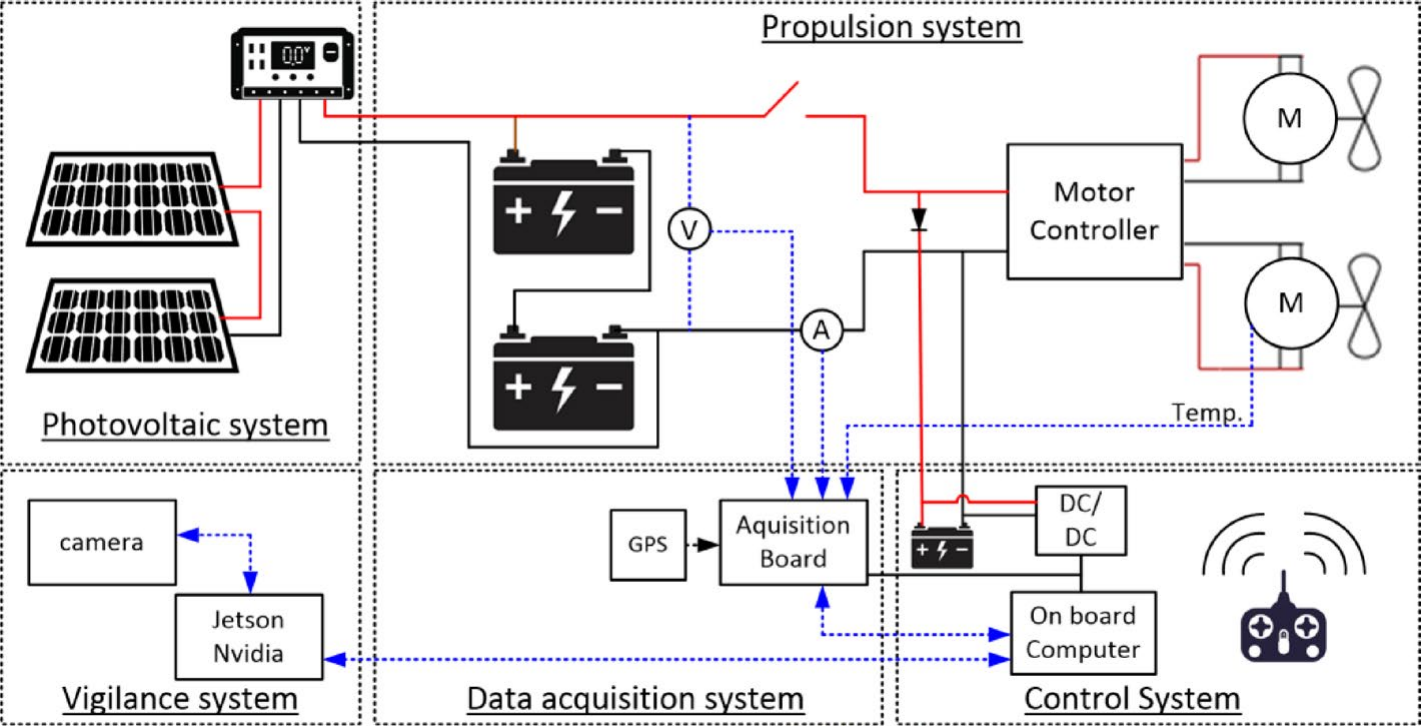
Electrical diagram of the USV.

The USV is divided into five main systems: the propulsion system, the control system, the data acquisition system, the vigilance system and the photovoltaic system. Each of these systems is divided into smaller and more specific sub-systems. In Fig. 4 the electrical diagram of all systems is shown.

### Propulsion system

The propulsion system is responsible for the movement of the vehicle and consists of the four following sub-systems:

- Batteries - Two Lithium-polymer batteries, connected in series, with a nominal voltage of 12 V, each one having a capacity of 100 Ah, a C-rate equal to 20, and a weight of 6.4 kg. The batteries are located in compartments 1 and 2, Fig. 3. Each battery is equipped with a battery management system.
- Motor Controller - The motor controller is capable of driving 2 DC motors, with a maximum supply voltage of 60 Vdc, a maximum continuous and peak current per channel of 60 A and 120 A, respectively, and a multi-input control mode, such analogue, PWM, serial or USB.
- DC Motors - Two 24 V DC electric motors, with a maximum rated current of 50 A per motor and a maximum force of 140 lbs and 1 kW mechanical power. The motors allow rapid mount/dismount, and easy adjustment of the direction, angle and depth, and are installed at stern.
- Propellers - Two preinstalled 2-blade propellers per motor, with a pitch of 4 inches and a diameter of 28 cm. Each set of motors and propeller is designed to perform a maximum thrust of 300 N.

### Control system

The control system is responsible for the control of the USV and can be divided into four sub-systems:

- On-board computer - One Raspberry Pi 3 model B+, running a Robot Operating System (ROS) on Ubuntu 20.04, with an additional USB WiFi dongle, for communication purposes. Also, the Raspberry Pi is connected to the instrumentation board using the General-Purpose Input/Output (GPIO) pins.
- RC Controller − 6 channel RC Controller with a Remote Transceiver (TX), and a Pulse Width Modulation (PWM) Receiver (RX). The Receiver is directly connected to the inputs of the Motor Controller, working as purely manual controller of the USV.
- Auxiliary battery - One small Lead-Acid battery, with a nominal voltage of 12 V and a capacity of 8 Ah. This battery is smaller than the main batteries since it is dedicated to the power of the on-board computer and dependencies. This battery is also charged by the main batteries when they are connected.
- DC-DC converters (24 to 12 V and 12 to 5 V) - Converts the 24 V from the main batteries to the 12 V needed to charge the auxiliary battery and power up part of the acquisition system and converts 12 V from the auxiliary battery to the 5 V needed to power up the on-board computer and part of the acquisition system.

### Data acquisition system

The data acquisition system is responsible for the acquisition of data from all the existing sensors in the USV. The main component of this system is a custom-made electronic board where the majority of the sensors are connected. Electronic circuits were designed for suitable signal conditioning and data transfer. This board is controlled by the on-board computer and connects to it using a 40-pin flat cable, providing power, and allowing data exchange.

The existing sensors in the USV are the following:

- Voltage Sensors – Six voltage sensing points are measured using a multi-channel of an Analog-to-Digital Converter (ADC). Upstream each channel, there is an adequate signal conditioning circuit consisting of resistive voltage dividers and RC filters. The sensing points of measurement are the 24 V from the main battery; the 12 V from the auxiliary output; and the positive and negative terminals of each motor, providing differential measurements for each motor. This ADC is present on the electronic board and the digital output is connected directly to the on-board computer via the flat cable. I2C protocol is used for bidirectional data communication.
- Current Sensors – Four magnetic current sensors with voltage output, connected to four channels of another ADC, through RC filters. These sensors measure the current provided by the main batteries, ranging from − 125 A to 125 A; the current provided by the solar panels/MPPT, ranging from 0 to 30 A; and the current demanded from each motor, ranging from − 62.5 to 62.5 A. The ADC is also present on the electronics board.
- Temperature Sensors - Two Negative Temperature Coefficient (NTC) sensors, connected in series with another resistance, working as a resistive voltage divider. The split voltage (temperature dependent) is connected to one of the previous ADCs. These sensors are being used to measure the temperature of two of the four compartments present in the hull of the USV.
- GPS - One GPS module, directly connected to the onboard computer via flat cable, retrieving NMEA GPS data messages, in particular the GPGGA and GPRMC messages. These messages are used to obtain different types of information, such as latitude, longitude and ground speed. To obtain a better signal, this GPS module also has an external antenna, which is fixed to the top of the hull.
- IMU – One Inertial Measurement Unit module, a 9-DOF sensor capable of measuring accelerations, orientation and angular rates.

### Vigilance system

A vigilance system composed of a Jetson microprocessor from Nvidea and a stereo camera ZED 2i was acquired and installed in the USV. The microprocessor is capable of handling video processing in real time, which is not achievable with a standard Raspberry Pi. The vigilance system is used to communicate video to a remote operator and to identify possible targets.

### Photovoltaic system

For this project, new photovoltaic panels and battery systems were selected to extend the current range of the USV mission. To take advantage of the maximum area of the USV and to avoid an excessive increase in the USV weight, four (4) flexible 100 W PV panels were acquired (Table 2). Considering the available area for installation, three PV panels were installed in the USV, and one was used for testing and redundancy. The three PV panels were installed in series to match the input voltage range from the DC converter. Preliminary tests to obtain its voltage-current curves were performed on the PV panels, in a laboratory and at real conditions, which confirmed the characteristics provided by the manufacturer.

To ensure the maximum extraction of the PV energy and control the charge of the batteries, a maximum power point tracker (MPPT) was installed between the PVs and the battery.

**Table 2:** PV characteristics.

Parameter	Value
Nominal power	100 W
Type of cells	Flexible monocrystalline
Nominal voltage	12 V
Open circuit voltage	21.45 V
Maximum power point voltage	18.15 V
Short circuit current	5.9 A
Maximum power point current	5.51 A
Weight	2.8 kg
Dimensions	1160 × 450 × 2 mm

### USV dynamic models

**Fig. 5:**
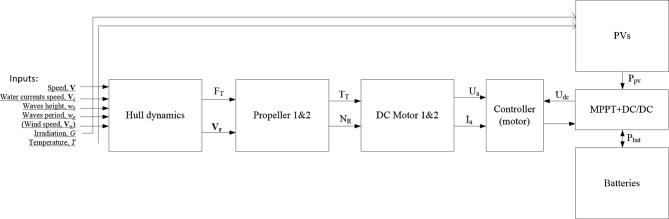
Proposed model of the USV.

This section concerns the modelling of the main subsystems of the USV that impact its energy consumption. Figure 5 shows the proposed white-box block diagram of the USV and its main subsystems’ models.

### Hull dynamics

**Fig. 6:**
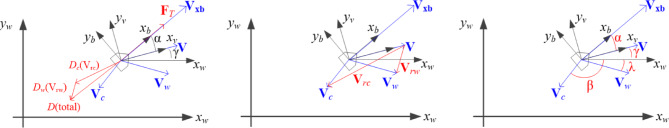
Full kinematic model of the USV.

The speed and force vectors are shown in Fig. 6. Axes (*x*_*w*_, *y*_*w*_), (*x*_*b*_,*y*_*b*_) and (*x*_*v*_, *y*_*v*_) correspond to the world, the USV body and the USV moving direction, respectively. Vectors **V**, **V**_**c**_ and **V**_**w**_ correspond to the USV, water current and wind speed directions, and **V**_**rc**_ and **V**_**rw**_ correspond to the relative speed between the USV and the water current and wind, respectively. Forces **F**_**T**_, **D**_**c**_, **D**_**w**_ and **D** correspond to the thrust, water current drag, wind drag and total drag vectors, respectively. Finally, angles *γ*, *β* and *λ* correspond to the angles between the world x-axis and the direction of the USV, the water currents and the wind, respectively, and *α* is the angle between the direction of the USV movement, *x*_*v*_, and the body orientation, *x*_*b*_.

With these, a kinematic model was developed for the USV, where the output of this function is the thrust and *α* angle required from the USV to perform the defined speed and direction, considering the water currents and wind.

**Fig. 7:**
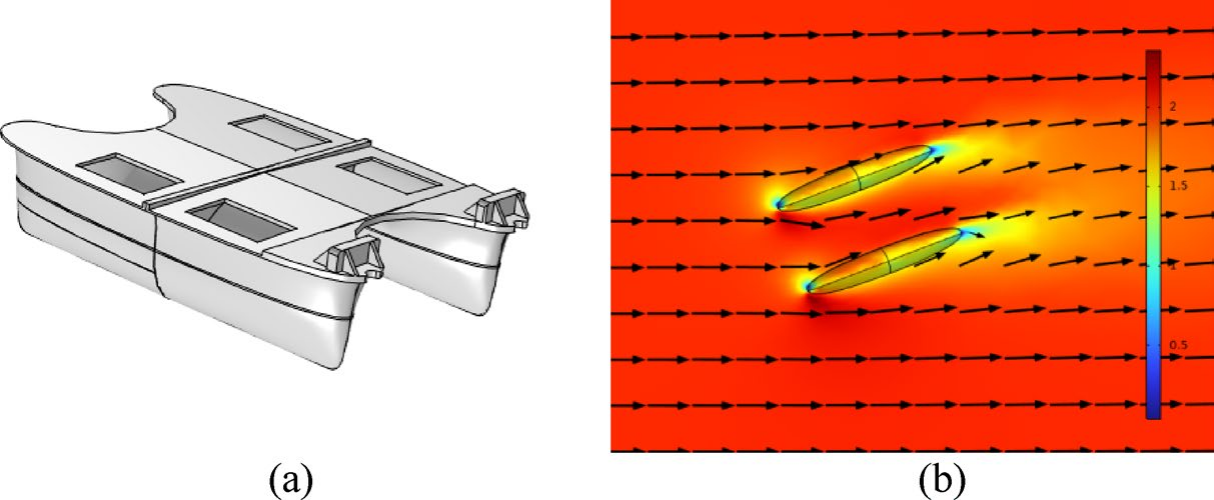
Finite Element model for the drag computation. In (**a**) the 3D USV model, and in (**b**) the water currents and intensity.

Due to the lack of analytical models to characterize the drag coefficients for a small size catamaran, it was decided to follow a hybrid approach with experimental results complemented by finite element results. Experimental tests were carried out to lay out the evolution of the drag coefficients with the water speed, which are further explained in “USV model validation”. A finite element model was also developed, in COMSOL Multiphysics, to complement the experimental data to further increase the models’ range, Fig. 7. This model considered a laminar and turbulent flow of the water currents flowing through different directions of the USV.

### Propellers and electric motors

**Fig. 8:**
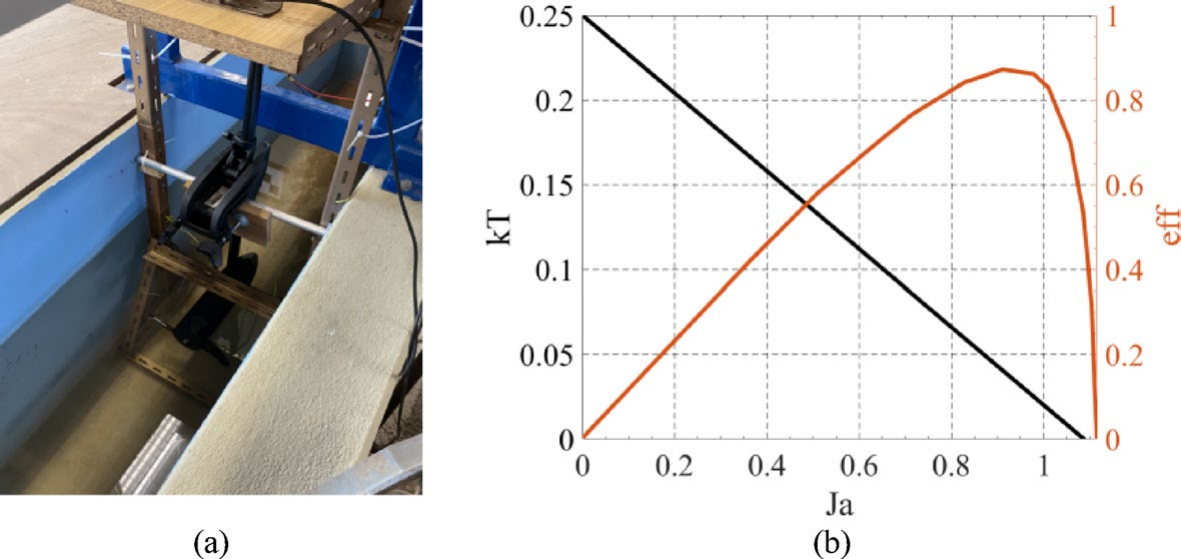
(**a**) Water channel for propeller experimental tests and (**b**) thrust coefficients and efficiency for the propeller.

The current USV propellers were characterized using experimental tests inside a water channel to obtain its torque-force curve and thrust coefficient *K*_*T*_, Fig. 8a. The thrust coefficient curve and propeller efficiency were estimated based on the experimental tests, Fig. 8b.

The DC PM motors of the current USV from this project were modelled and validated through experimental tests. No-load, locked-rotor and load tests were performed to obtain the motor’s main electric and mechanical characteristics. In Table 3 are presented the motor’s equivalent electric circuit parameters, where *R*_*a*_ and *L*_*a*_ are the armature resistance and inductance, *k*_*ϕ*_ and *k*_*h*_ are the constants associated to the electromotive force and hysteresis losses, *β* and *J* are the viscous friction and inertia of the rotor.

**Table 3:** Electric motors’ main characteristics.

*R*_a_ [Ω]	k_ϕ_ [Wb]	k_h_ [W$\:\cdot \:$s]	$\:\beta\:$ $\:[\text{N}\cdot \:\text{m}\cdot \:\text{s}]$	L_a_ [H]	J [kg$\:\cdot \:$m^2^]
0.176	0.0907	0.1802	9.9 × 10^− 4^	8.97 × 10^− 4^	7.24 × 10^− 4^

The motor model was developed for convenience with the voltage, *U*_*a*_, and torque load, *T*_*L*_, as input and the motor current, *i*_*a*_, and speed, *N*_*r*_, as output, as can be seen in Eq. (1).

1 $$\:{i}_{a}=\frac{1}{{L}_{a}}\int\:{U}_{a}-{i}_{a}{R}_{a}-{k}_{\varphi\:}{\omega\:}_{r}dt{\omega\:}_{r}=\frac{\:1}{J}\int\:{k}_{\varphi\:}{i}_{a}-{T}_{L}-\beta\:{\omega\:}_{r}-{k}_{h}dt$$

### Batteries and photovoltaic panels

**Fig. 9:**
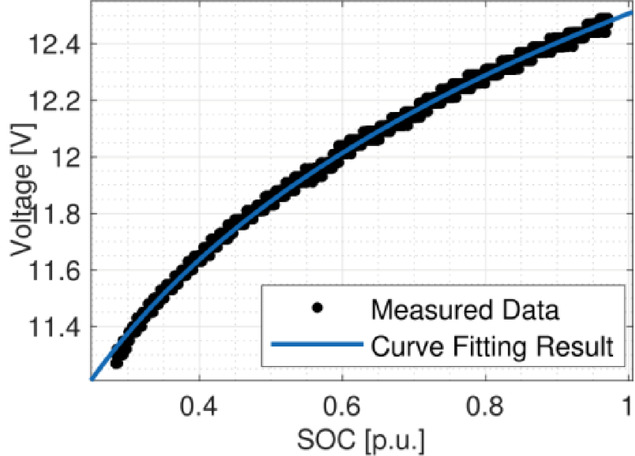
Variation of V_oc_(_SOC_). Experimental results are in black dots, and the fitted equation is in blue line.

Lithium-phosphate (LiPo) 12 V batteries were installed on the USV with a total of 2400 Wh and 30 kg of weight. The batteries can be modelled using a typical equivalent electric circuit. However, due to the large mission times, their electric transients became negligible. Therefore, only the evolution of the open-circuit voltage (*V*_*oc*_) with the stage of charge (*SOC*) and its internal resistances were used to model it. The *V*_*oc*_(*SOC*) was obtained experimentally and fit with $\:{V}_{oc}\left(SOC\right)=12.47\cdot \:{SOC}^{0.0995}$, Fig. 9, and their internal resistance was calculated, *R*_*0*_ = 10 mΩ.

### Photovoltaic panels

For a good estimation of the available energy production from solar cells, Eqs. (3) and (4) have been proven to be sufficient under calm weather conditions^37^. The temperature of the solar cells was estimated from (2), using the ambient temperature, *T*_*amb*_, and irradiation, *G*, and the energy production was calculated from (3), using the solar panel STC power, *P*_*STC*_, and temperature coefficient, $\:{\alpha\:}_{p}.$

2 $$\:{T}_{PV}={T}_{amb}+G(NOCT-20)/800$$

3 $$\:{P}_{PV}={P}_{STC}\frac{G}{1000}(1+{\alpha\:}_{p}\left({T}_{PV}-25\right)$$

## USV model validation

**Fig. 10:**
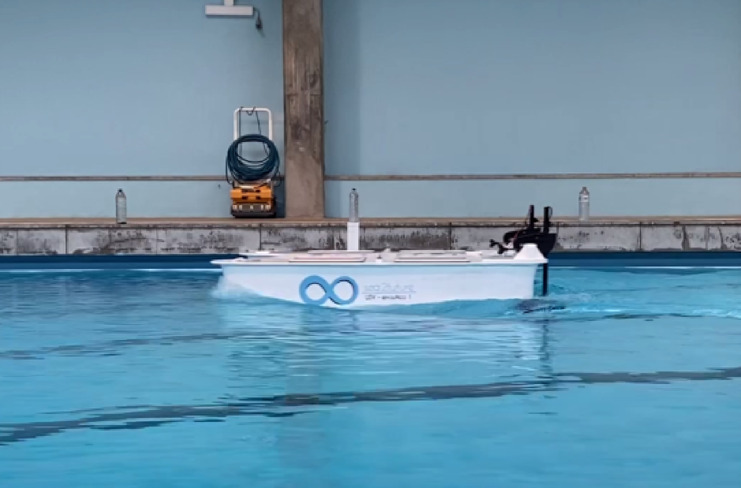
Photos of the experimental environments: swimming pool.

**Fig. 11:**
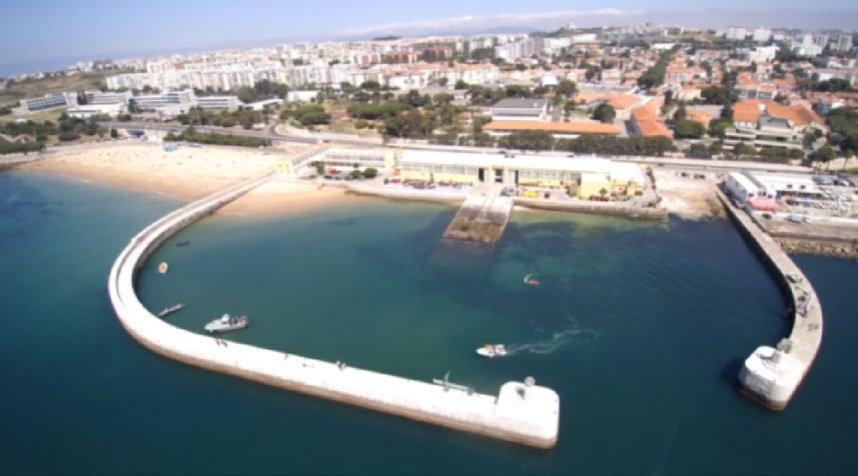
Photos of the experimental environments: enclosed harbour.

The USV model validation was achieved in the previously published work^36^. Experimental tests were performed in an indoor swimming pool, Fig. 10, and in an outdoor enclosed harbour at Tejo river, Fig. 11. A summary of the results is presented in this section.

The pool tests allowed for underlying the USV behaviour without wind, water current and waves, while the enclosed harbour tests allowed a more realistic context. With these, the hull and propeller models were calibrated to be further used in the optimization scenario.

Based on the experimental tests, the USV model was calibrated and verified. Please note that, for its calibration, the speed of the USV and the current and voltage of the batteries and electric motors were recorded during the experimental test. It was decided to use as input variables of the model the batteries’ current and voltage and verify the model against the experimental USV speed.

**Fig. 12:**
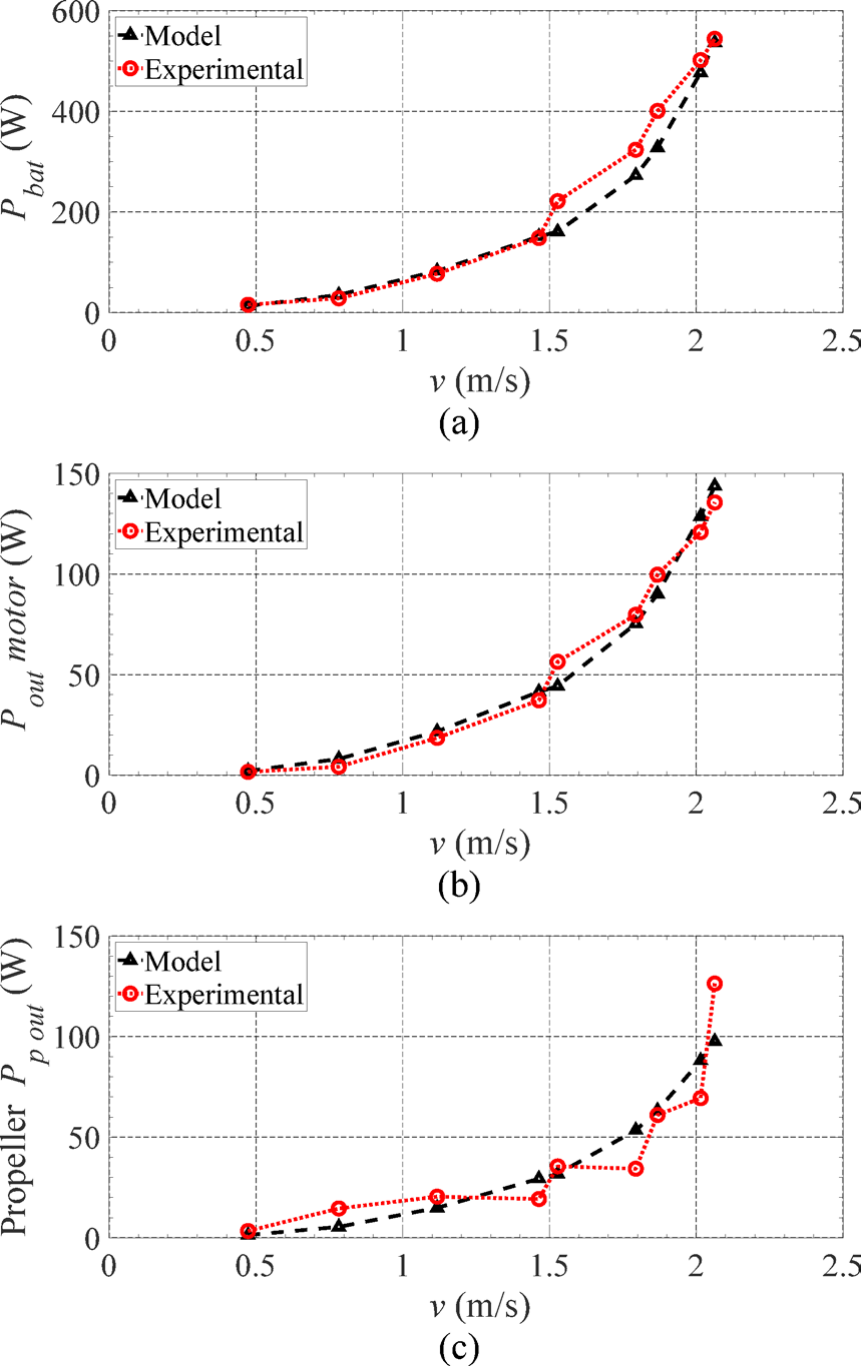
Experimental and simulation results: (**a**) battery output power, (**b**) electric motors output power, and (**c**) propellers output power^36^.

Figure 12a–c show the indoor experimental and simulation output power results, for the batteries, motors and propellers at a constant speed. To obtain the simulation results, the total drag coefficient was calibrated based on experiments. As expected, the required power is proportional to the cubic of the speed. The model results fit well the experimental ones in all three cases.

**Fig. 13:**
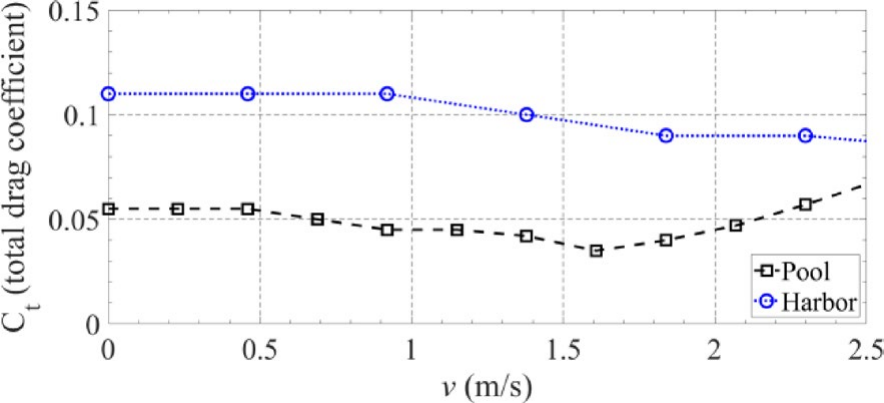
Total drag coefficient obtained experimentally.

**Fig. 14:**
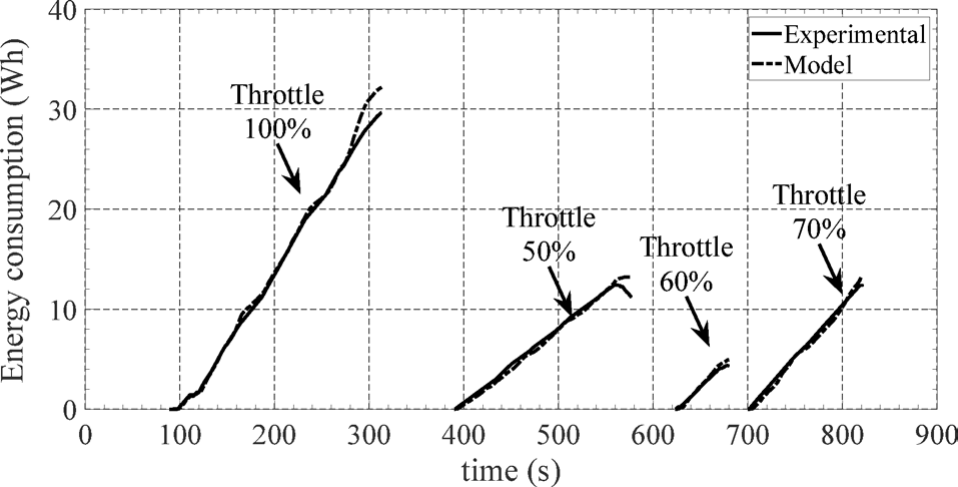
Experimental and simulation results for the energy consumption at the different throttle levels^36^.

The enclosed harbour tests were performed at the Tejo river, Lisbon. These allowed for estimating the increase of the drag due to the presence of waves. When compared with the indoor tests, an increase in the total drag coefficient was verified (Fig. 13). For lower values of speed the drag coefficient doubled its value but converged for values close to 2.5 m/s. Figure 14 shows the experimental and simulation results for the USV accumulated energy consumption in the harbour, with different values of throttle percentage to its maximum value. From the energy point of view, the results achieved clearly indicate that the developed USV simulation model is capable of predicting accurately the energy consumption for all different throttle values applied to the motors. Therefore, one can have confidence in the developed USV simulation model to perform the optimization scenarios and carry out the experimental tests with the developed SEMS.

## Smart energy management system

The smart energy management system is divided into three steps: (1) meshing of the mission area, (2) path optimization at a constant speed, (3) speed optimization for the optimal path.

A Bidirectional Graph-based algorithm (BdG) and a new A-star-based algorithm with probabilistic behaviour (A*Pb) were used for the path optimization. The Graph-based algorithm considers constant environmental conditions along the mission and, thus, it is only suitable for form mission durations. The new A-start-based algorithm with probabilistic behaviour was developed, which presented the capacity to handle time-variable environmental conditions, being suitable for long mission durations. In this section, the proposed algorithms will be presented and discussed.

**Fig. 15:**
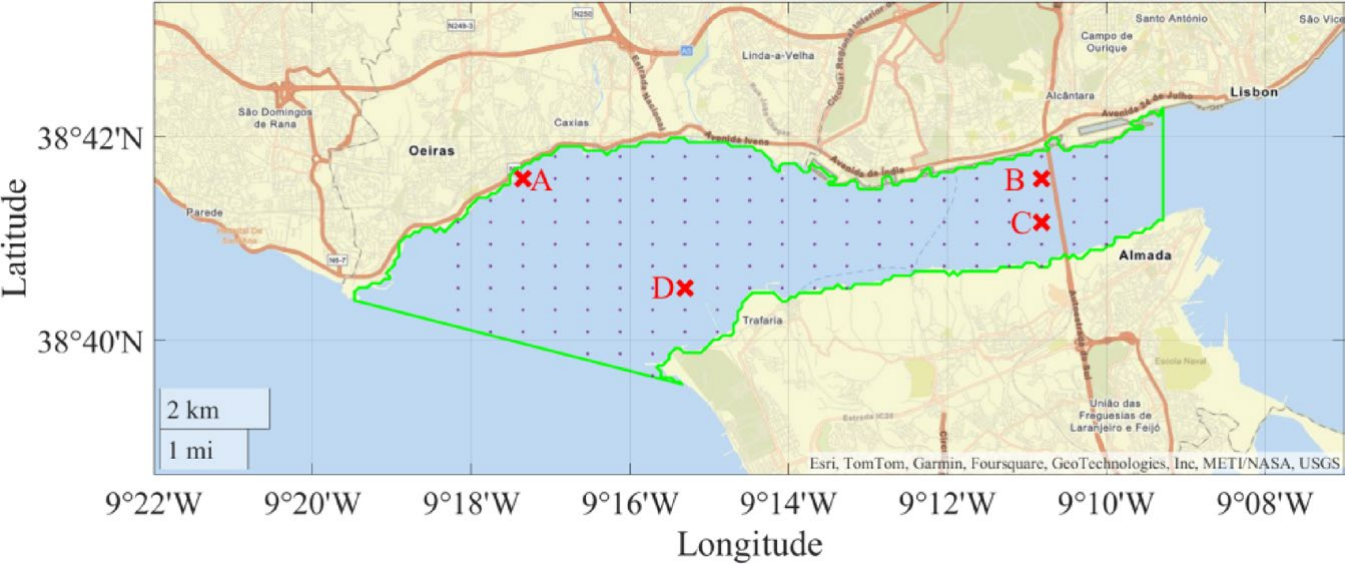
Mission map.

The path optimization was performed using a grid of points distributed along the river, as shown in Fig. 15. The real distance between points is 600 m along the longitude and 450 m along the latitude. However, these distances can be redefined by the mission planner. The mission was carried out in the mouth of Tejo river, in Lisbon, limited by the green line. The USV mission should be carried out by starting on point A (ENIDH location), crossing points B, C, and D, in this order, and finally ending again on point A (A -> B -> C -> D -> A). The black points create a searching grid where the USV can move.

### Bidirectional graph-based algorithm (BdG)

A bidirectional graph was constructed with all the possible paths between points, where the edges between two points are represented by weights. The weight definition corresponds to the energy required by the batteries between two points, considering a constant speed and the USV direction. For the BdG algorithm, these consider static environmental conditions recorded at the beginning of the mission. The final solution is optimum, finding the global minimum energy path, using a bidirectional shortest path algorithm.

Considering its characteristics of static environmental conditions, this approach is only suitable for short-time missions, which was the reason for the development of a new A-star algorithm. However, for these short missions, the BdG algorithm is capable of finding the optimal global solution in a short time. The results of this BdG algorithm were presented previously at the MELECON2024 conference^36^.

### A-star based with probabilistic behaviour (A*Pb)

**Fig. 16:**
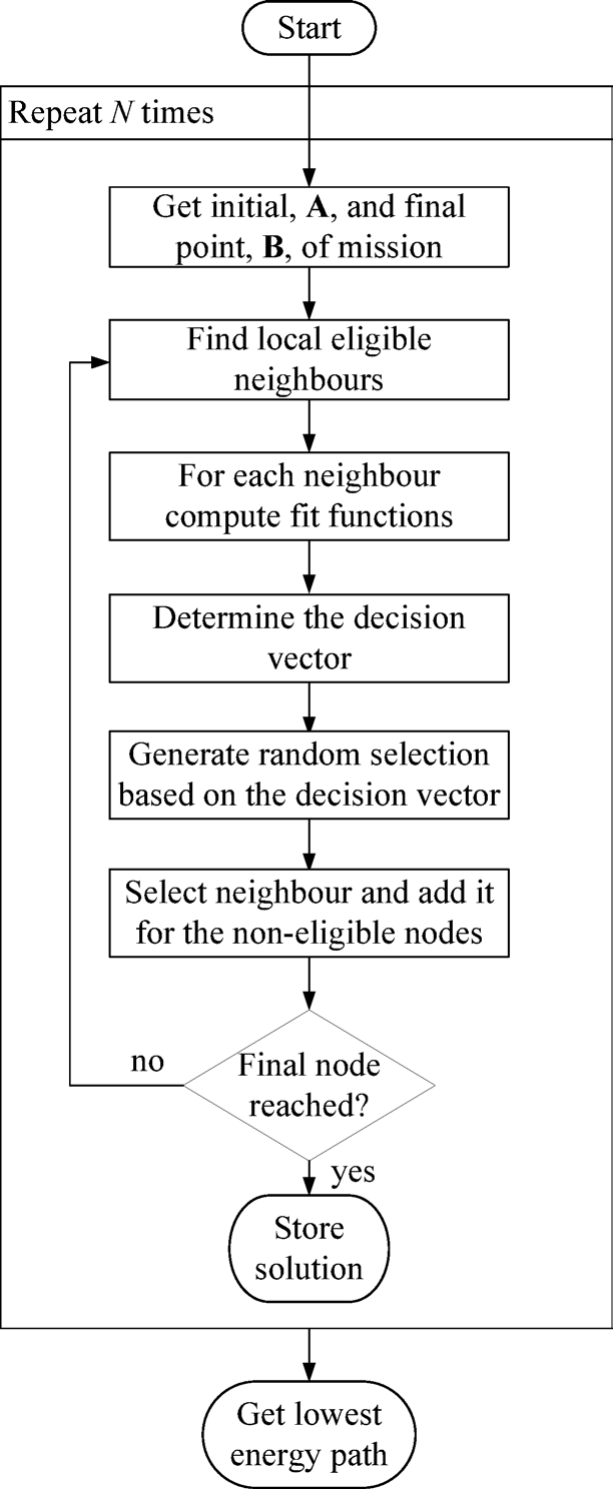
Flowchart of the A*Pb algorithm.

The graph approach is not suitable when considering time-varying environmental conditions, because the weight between two nodes is not constant, leading to infinite solutions. Therefore, for long-time missions, it is not possible to use a graph approach. The solution proposed to handle this problem is the A-star-based algorithm with probabilistic behaviour (A*Pb). This was inspired by the A-star algorithm and on genetic-algorithm optimization methods. Genetic algorithms typically use probabilistic behaviours, such as crossovers and mutations, to avoid getting stuck at local minimum solutions, while searching for an optimal solution. We decided to implement a A-star based algorithm for the path optimization because the A-star algorithm is typically fast, more efficient than Dijkstra (for example) when search space increases, and with low computational cost. In addition, we included a probabilistic behavior to better suit the unpredictability of the environmental conditions changes along the mission and avoid local minimums. The flowchart and pseudo-code of the proposed A*Pb is shown in Fig. 16; Table 4.

**Table 4:**
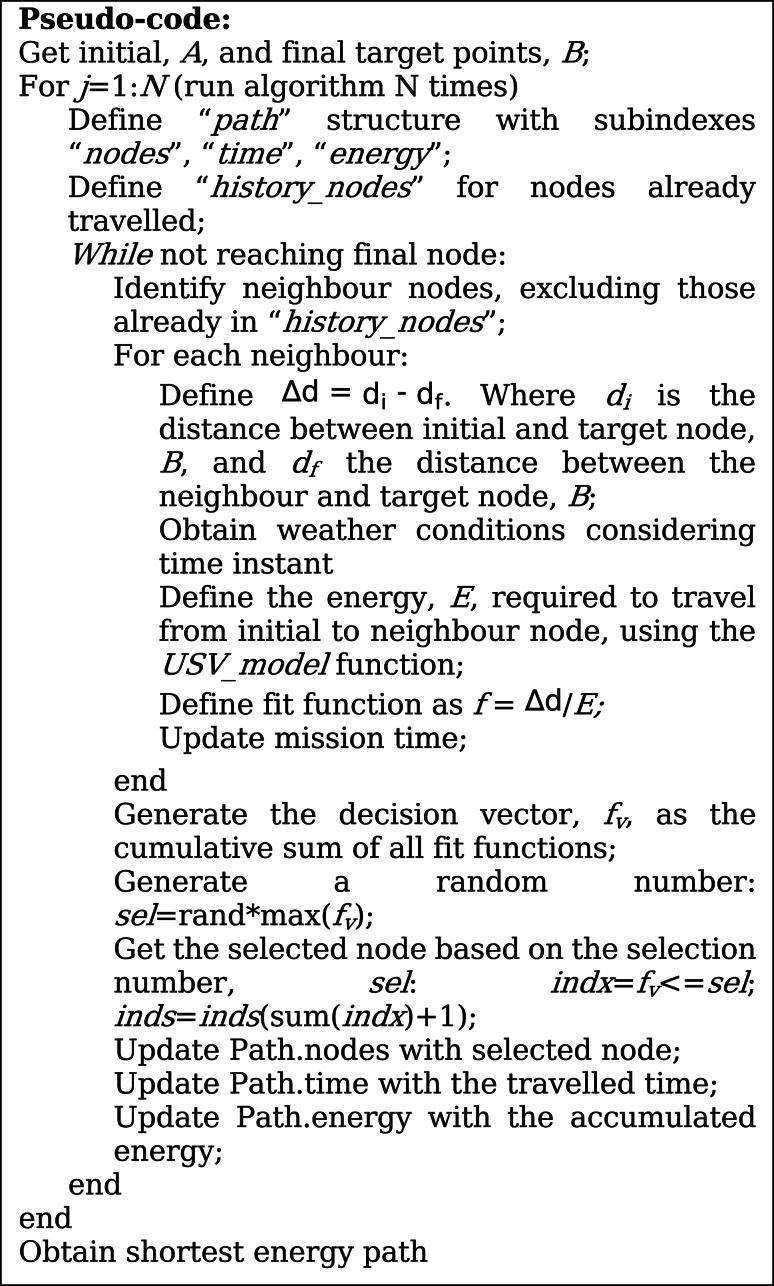
Pseudocode of A*Pb algorithm.

In summary, the algorithm starts by defining the initial, A, and target, B, nodes. Then, an iterative process was followed until the target node was reached. The process starts by obtaining the neighbour nodes of the initial node and excluding the nodes that have already travelled (*history_nodes*). For each neighbour node, the fit function *f* was obtained by dividing $\:\varDelta\:d$ by *E*. The parameter $\:\varDelta\:d$ corresponds to the difference between the distance from the initial to the target node, *d*_*i*_, and the distance between the neighbour node to the target node, *d*_*f*_. The energy, *E*, required to travel between the initial and the neighbour nodes was obtained by the USV model. This process was done for all neighbour nodes. After the definition of the fit function of all neighbour nodes, a decision vector, *f*_*v*_, was built by performing the cumulative sum of all fit functions. For example, if it was obtained *f*_*1*_ = 10, *f*_*2*_ = 5 and *f*_*3*_ = 1, then the decision vector is defined as *f*_*v*_=[0 10 15 16]. Then, a random number, *sel*, is generated between the interval of the decision vector, [0 16] for the previous example, and selecting the neighbour node based on it. For the example shown, if the generated number is *sel* = 5, then it falls within the [0 10] interval, corresponding to neighbour 1. If the generated number is 13, then it falls within the second interval [10 15] corresponding to neighbour 2.

The idea of this random process was to generate a number with a higher probability of finding the best-fit function, however, it has a non-null probability of selecting another non-local-optimal node. This allows the algorithm to not get stuck in the local optimum and find alternative paths that may be better in the future due to the change in environmental conditions.

This algorithm was performed *N* times, each time finding an optimal path and, in the end, the path with the lowest energy consumption was selected. The *N* variable is defined by the user.

### Simulated performance under typical mission scenarios

**Fig. 17:**
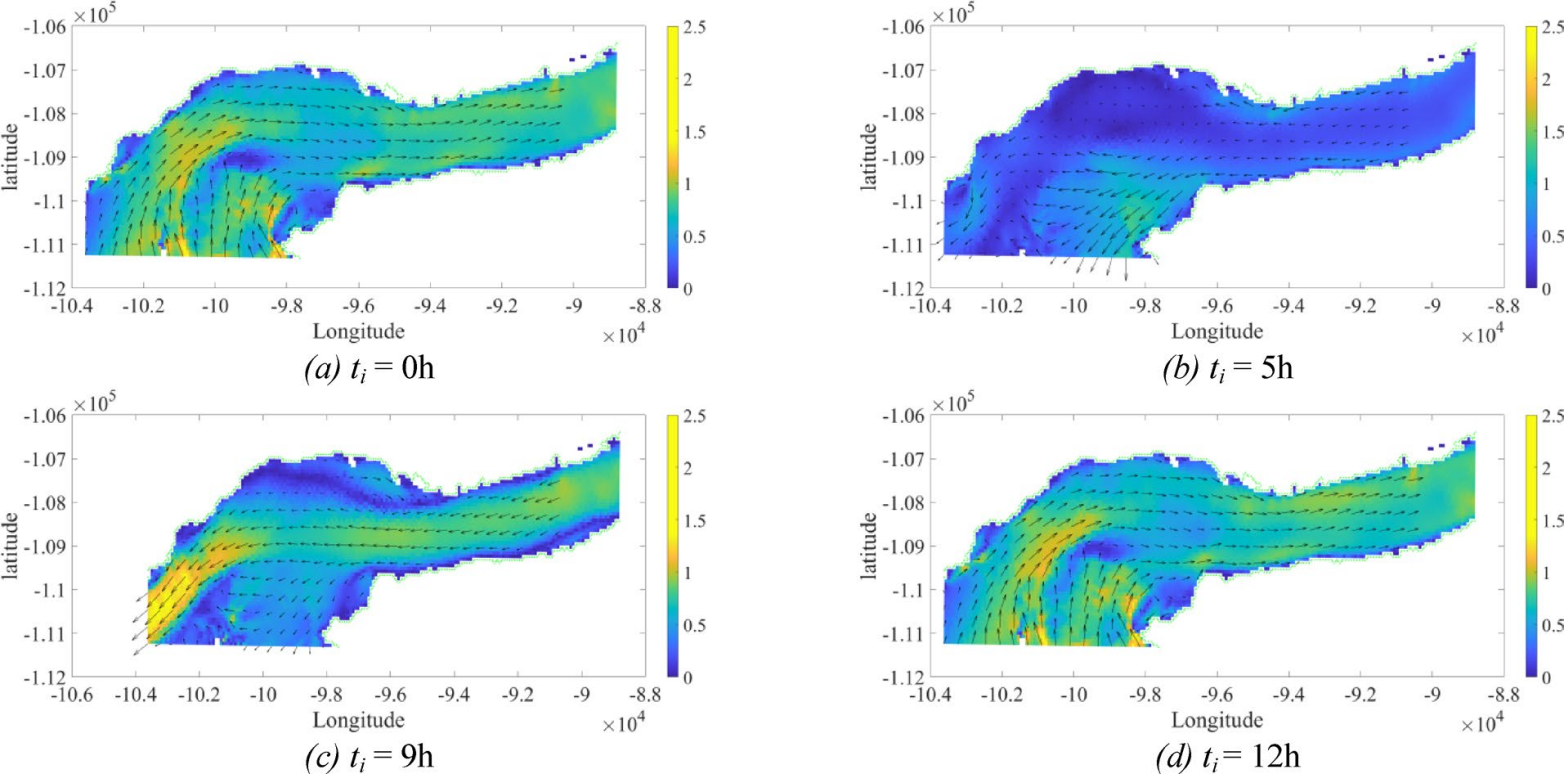
Water current map for the different initial local times: (**a**) *t*_*i*_ = 0 h, (**b**) *t*_*i*_ = 5 h, (**c**) *t*_*i*_ = 9 h and (**d**) *t*_*i*_ = 12 h. The colour map corresponds to the magnitude of the water currents in m/s and the arrows present the current’s directions.

Both SEMS algorithms are now simulated and compared under the case study of the surveillance mission using the grid points despited in Fig. 15. The proposed algorithms will select the best path and speed profiles to meet the mission requirements. The optimal path, leading to the minimum energy consumption will depend on the environmental conditions, such as water currents directions and magnitude. Because the mission was carried out on Tejo river, the water currents present a cyclic behavior, between ebb and flood conditions, at different local times for different days of the year. To test the proposed algorithms’ performances, the mission perfomance was computed with different initial local times equal to *t*_*o*_ = [0 5 9 12] h, with results shown in Table 5. This was done to test the algorithms’ resilience to different environmental conditions. Figure 17 shows the water current maps at the different initial local times: (a) *t*_*o*_ = 0 h, (b) *t*_*o*_ = 5 h, (c) *t*_*o*_ = 9 h, and (d) *t*_*o*_ = 12 h. The colour map corresponds to the magnitude of the water currents and the arrows present the current’s directions. It can be seen that the water currents have a period of around 12 h, and thus, it is expected for them to change directions each 6 h.

### Comparison between algorithms

The performance of the bidirectional graph (BdG) and the A-star algorithm with probabilistic behaviour (A*Pb) are presented in Table 5. Because of its limitations, the BdG algorithm does not allow to consider time-variable environmental conditions during its path optimization. Therefore, the BdG algorithm performs its optimization considering static environmental conditions equal to the ones at the mission initial time (“Predicted Energy” in Table 5). After the selection of the BdG optimized path, the same path is then recalculated using the time-variable environmental conditions (“Corrected Energy” in Table 5) and the deviations are shown (“Deviation” in Table 5). The A-star algorithm is able to handle time-variable environmental conditions, thus, no correction is required. The energy ratio (ER) between the A-star and BdG algorithms is shown Table 5.

Based on the results it is possible to understand that the BdG algorithm, considering the initial weather conditions, follows the path where the initially water currents are favourable. However, due to the long mission times, this does not always lead to a good option. For example, for *t*_*i*_ = 5 h, the weather conditions change drastically during the mission and the corrected energy consumption is 5.19 kWh, which is 152% times higher than the initial predicted one. On the opposite, the A*Pb algorithm is able to find a path with a minimum energy of 3.05 kWh, about 41% lower than the one performed by the BdG algorithm.

**Table 5:** Performance comparison between algorithms.

Initial time	Bidirectional Graph-based algorithm (with static environmental conditions)				A-star algorithm with probabilistic behaviour (with time-variable environmental conditions)		Energy Ratio between A-star and BdG Algorithms (*E*$\:ER=\frac{{A}_{star}}{BdG}-1$)
	Predicted energy	*Corrected energy	Deviation	Mission time	Calculated energy	Mission time	
0 h	2.37 kWh	2.01 kWh	– 15%	7,0 h	2.01 kWh	7,8 h	+ 0%
5 h	2.06 kWh	5.19 kWh	+ 152%	6,3 h	3.05 kWh	7,5 h	– 41%
9 h	1.64 kWh	3.49 kWh	+ 113%	6,4 h	3.09 kWh	7,4 h	– 11%
12 h	2.37 kWh	2.04 kWh	– 14%	7,0 h	2.04 kWh	7,7 h	+ 0%

*Corrected energy is computed using the path chosen by the BdG algorithm with constant conditions but recalculated using variable conditions.

The BdG, with constant sea conditions, constantly fails to predict the energy consumed. Sometimes it even overestimates the energy required, as is the case with missions that start at 0 h and 12 h. In addition, when the sea conditions are more favourable for the first moment of the mission, it underestimates the energy required too much (cases of mission time starting at 5 h and 9 h).

The new A*Pb algorithm considers actual sea conditions to vary over time. Of course, it is not able to guarantee the overall minimum, however, as it is run several times (following a Monte Carlo logic), it is always able to obtain a required energy equal to or lower than that of the previous algorithm. In cases where sea conditions start favourable (5 h and 9 h), it is able to find paths that avoid areas where sea conditions change from favourable to unfavourable. The obtained mission times are also not much higher than the initial algorithm and are sometimes even lower.

With this, it can be concluded that the proposed Smart Energy Management System is suitable for short (BdG) and long missions (A*Pb).

Note that the initial algorithm takes about 30 s to calculate a mission, while the new algorithm takes about 3 to 5 min, using a laptop with 16Gb of RAM memory and an Intel Core i7-6700 processor. However, as the mission is to be priori planned, this increment of time is not significant.

## Experimental results

**Fig. 18:**
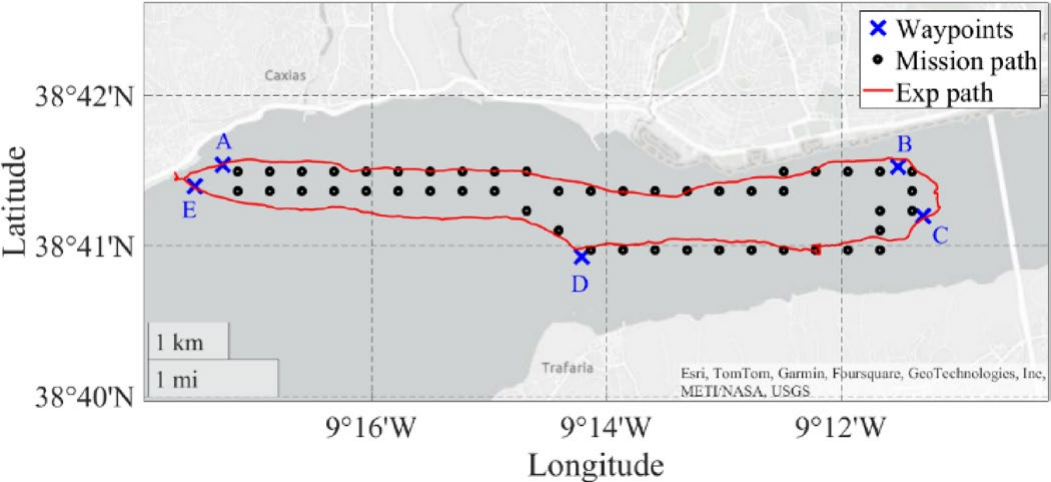
Experimental mission carried out at Tejo river, Lisbon, Portugal.

The final experimental tests were carried out at Tejo river, Lisbon, Portugal, at the interface between the Atlantic Sea and Tejo river, Fig. 18. The mission consisted of starting from the harbour at *Paço de Arcos* (waypoint A), travelling until the bridge “*Ponte 25 de Abril*” (waypoints B and C), following to *Trafaria* (waypoint D) and returning to the harbour (waypoint E). The mission was carried out on the 17th of July 2024, starting at 10 h and 30 min local time.

In Fig. 18 are presented the mission waypoints, the mission path points in “•” defined by the SEMS algorithm and the experimental points measured from the GPS of the USV. The total mission time was about 3 h and 45 min, from which 3 h 15 min corresponded to the mission itself, and the last 30 min to the preparation of the USV removal from the harbour.

**Fig. 19:**
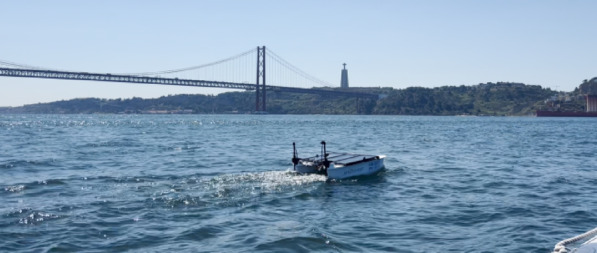
Photo between waypoints B and C.

It is worth mentioning that, during the mission, some water droplets fell on top of the photovoltaic panels, however, no negative impact was verified in their power production. In fact, the power production was slightly higher than expected due to the cooling action of these water droplets. At the end of the mission, due to the evaporation of these droplets, salt remains were visible on the top of the panels. Note that, the mission was carried out on the interface between the sea and Tejo river, and thus, with the presence of salted water. However, it was easily removed with fresh water after the end of the mission (Fig. 19).

### A*Pb computational efficiency

**Fig. 20:**
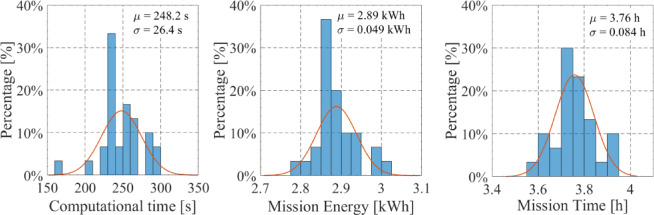
A*Pb algorithm computational efficiency for the experimental mission.

Regarding the computational efficiency of the A*Pb algorithm, due to its probabilistic behavior, its computational time also presents a probabilistic behavior. Therefore, to assess its efficiency, the path optimization of the experimental mission was recalculated 30 times, each one with *N* = 100 iterations. The results concerning the calculation of the full mission are presented in the Fig. 20; Table 6. The measurement of average values and standard deviations is not trivial mainly due to the non-linearty of the models and the dynamic environmental changes. After the trials, however, the results, shown in Fig. 20, tend to show a Gaussian distribution behaviour, thus with a Gaussian distribution fit average values and standard values were estimated.

As can be seen, the computation time required to optimize the whole mission presents an average value of 248.2 s and a standard deviation of 26.4 s. Regarding the mission energy and time their standard deviations are very low when compared with the mean value, which indicates a good performance of the algorithm. Considering an average value of around 250 s of required computational time, and an USV speed of 2 m/s, the mission can be optimized every 500 m, which corresponds to about 2.5% of the mission range (around 20 km). If the USV speed is decreased to 1 m/s, the proposed algorithm would be able to ensure an optimized path and speed, including unpredicted environmental changes, every 1.25% of the mission range, i.e., every 250 m. By comparison, in^38^, the authors state that they are able to plan the next optimal path for an UAV, using particle swarm optimization, every 1700 m. Our algorithm is able to perform the path optimization every 250–500 m. In^39^, the authors designed a real-time path planning for autonomous UAVs, achieving computational times of around 100 s for a path within a 2D plane of 10 km per 10 km. Because the searching space is 2D, an increase from 10 km by 10 km to a 20 km by 20 km search space would lead to an increase of computational time between two (linear dependency) to four times (quadratic dependency), reaching computational times between 200 and 400 s. In comparison, our A*Pb algorithm requires similar computational times. In^40^, the authors use different path optimization algorithms for a UAV, achieving the best computational time of 1.2 s for a path length of 66 m, leading to around 360 s for a total of 20 km. These examples show that our proposed strategy is suitable for these types of applications.

**Table 6:** Performance of the A*Pb algorithm.

Parameter	Average value	Standard Deviation
Computational Time [s]	248.2	26.4
Mission Energy [kWh]	2.89	0.049
Mission Time [h]	3.76	0.084

### Mission results

**Fig. 21:**
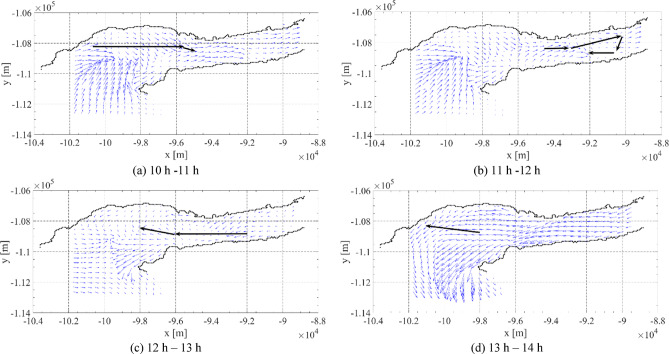
Evolution of predicted water current together with the mission path. USV path indicated as black arrows.

To carry out the mission, the SEMS A*Pb algorithm was used a-priori with the predicted environmental conditions, starting at 10 h and 30 min local time on the 17th of July 2024. The algorithm was set with a USV speed of 2 m/s that would lead to about 20% of the battery energy at the end of the mission, for safety. Figure 21 shows the predicted environmental conditions at different hours of the day and the carried out USV path indicated as black arrows. Note that the mission starting time was chosen so that the water currents were favourable along the mission, as predicted by the A*Pb algorithm. The currents were from west to east during the first part of the mission (10–12 h), and from east to west during the second part of the mission (12–14 h).

During the mission, the following data was acquired in real time:

- GPS: time, latitude, longitude, speed overground, direction, number of satellites.
- Sensors: time, voltage and current of motors 1 and 2, batteries and photovoltaic panels, and motors’ temperature.

**Fig. 22:**
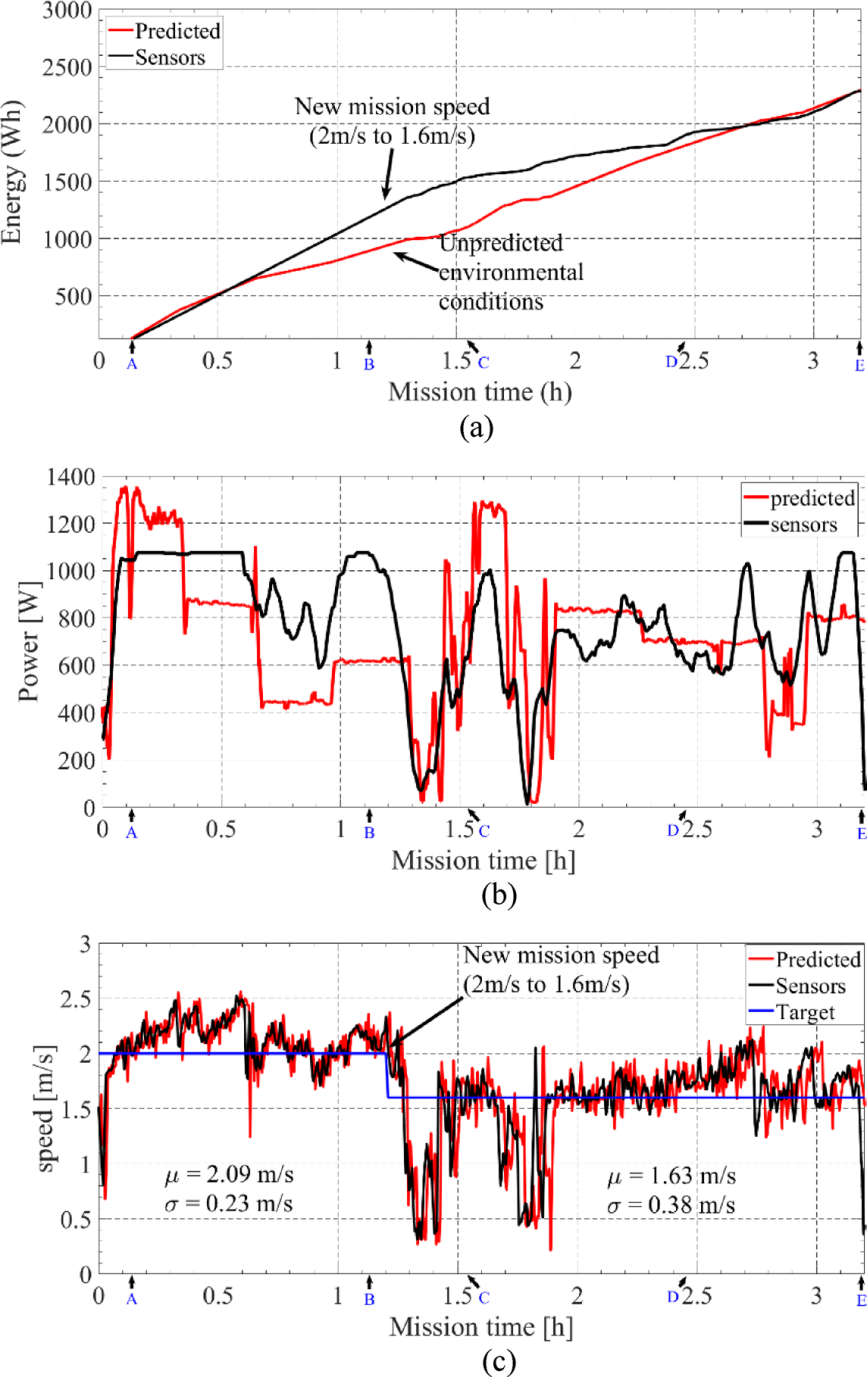
Evolution of predicted and measured (**a**) accumulated energy consumption, (**b**) instant power and (**c**) target and measured speed.

Figure 22 shows the evolution of predicted and measured accumulated energy consumption and target and measured speed. The final measured energy consumption was 2.31 kWh and the predicted one was 2.28 kWh, presenting a deviation less than 1.5%.

As can be seen between 0.5 h and 1.2 h, the measured energy consumption presented a deviation due to the unpredicted increase of waves. This increase would compromise the mission if the same USV speed was maintained. This effect can be clearly seen in Fig. 22b, where the measured power (sensors) is higher than the predicted required power between 200 and 400 W. Therefore, at 12 h, corresponding to the waypoint B, it was decided to optimize the energy planning by changing the target speed that would lead to a remaining of 20% of battery energy at the end of the mission.

**Fig. 23:**
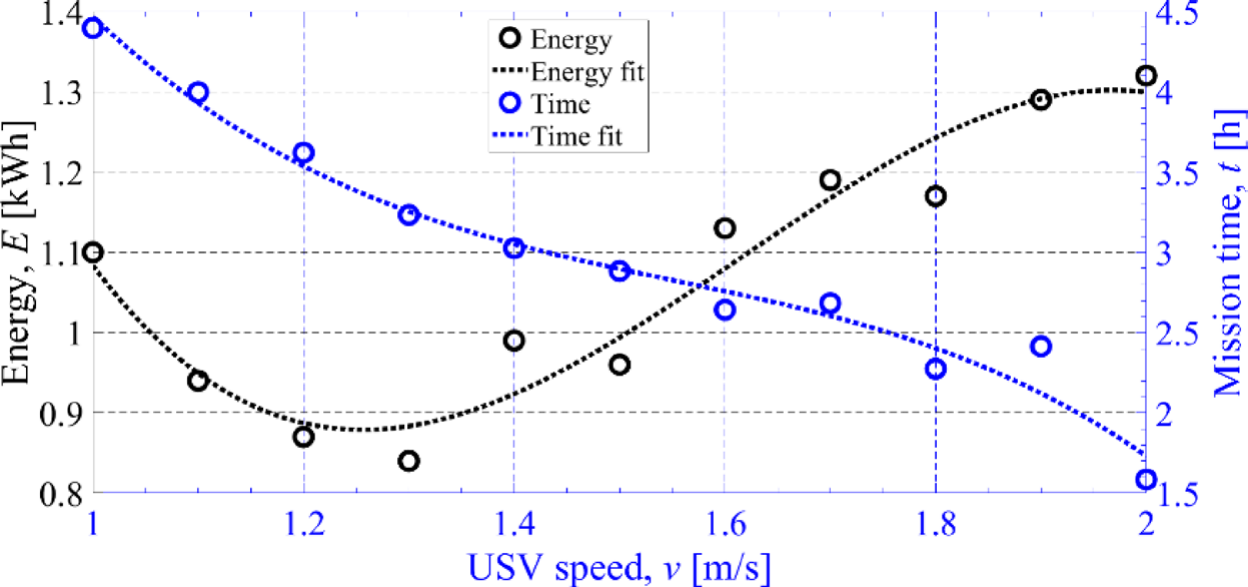
Impact of USV speed on the energy required to carry the remaining of mission from waypoint B to E. The selected speed is 1.6 m/s, originated from the intersection between the two curves.

When arriving at waypoint B, the remaining useful energy stored in the batteries, considering a backup of 20%, was about 1.1 kWh. With the additional predicted photovoltaic available energy of about 0.4 kWh, a total of 1.5 kWh of useful energy was available to carry out the rest of the mission. Therefore, the A*Pb algorithm was recalculated with different target USV speeds, and the obtained results are shown in Fig. 23. As it can be seen, to achieve the 1.5 kWh of energy the speed should decrease to about 1.6 m/s. As a result, the target speed was changed from 2 m/s to 1.6 m/s after 1.2 h of mission time, Fig. 22b.

The measured USV speed presented a mean value of about 2.09 m/s with a standard deviation of 0.23 m/s from waypoint A to B and a mean value of 1.63 m/s and a standard deviation of 0.38 m/s for the remaining part of the mission. Considering their mean values, the deviation to the target speed was about 4.5% between waypoints A and B and 1.9% between waypoints B and E.

**Fig. 24:**
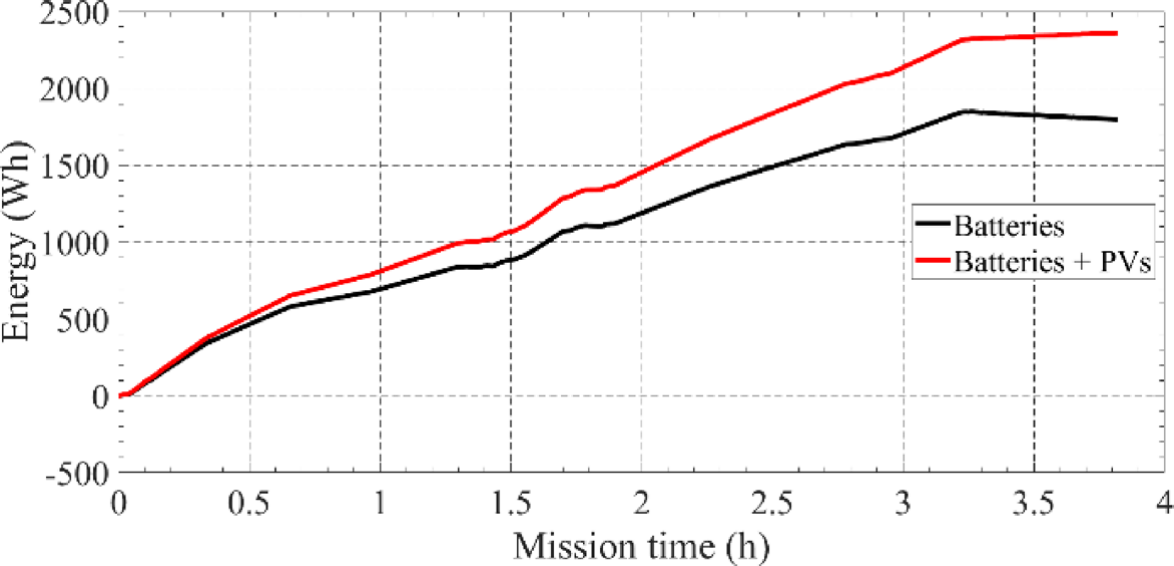
Energy distribution between the battery and photovoltaic panels.

The mission was carried out with success, finishing in 3.2 h, and with a remaining battery energy of 20%. During the last 30 min of the mission, the USV was stationary at the harbour, waiting to be removed from the water. During this time, the PV panel charged the batteries 3%. This can be seen in Fig. 24, showing the accumulated energy consumed from the batteries and photovoltaic panels. After achieving the final waypoint E, at *t* = 3.24 h, the accumulated energy consumed from the battery accounted for *E* = 1846 Wh. After approximately 30 min, with the USV stationary at the harbor, the final energy from the batteries achieved *E* = 1795 Wh, about − 3% less. This means that the PV panel charged the batteries, i.e., with negative battery output power, in about 50 Wh, 3% of the accumulated final at the end of the mission.

**Fig. 25:**
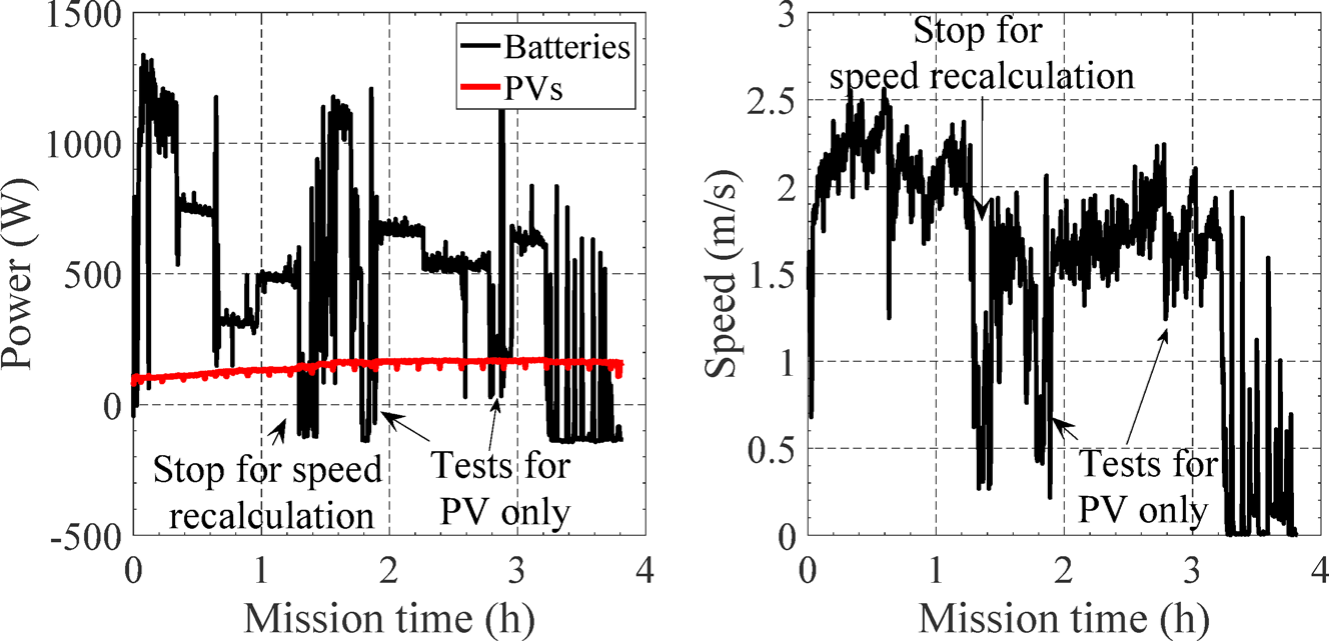
Source power distribution and USV speed along the mission.

To further analyse the importance of the PV panels, the measure of energy distribution along the mission is shown in Fig. 24. The PV panels produced 350 Wh, which corresponds to an increase of 21% in energy availability. These 21% were critical to ensure a final battery energy of 20% at the end of the mission. Note that the mission time was relatively short (about 3.2 h), therefore, the PV energy was limited. If the mission was carried out at 1 m/s, a further increase of 40% in energy would be expected from the PVs. Furthermore, during the mission it was tested the possibility of operating the USV only by the PV energy (without batteries), this was achieved at different stages of the mission, with USV speeds between 0.5 m/s and 1.2 m/s, Fig. 25.

### Comparison with the initial USV prototype

After carrying out the mission, the influence of each proposed subsystem was verified. As stated before, the inclusion of the PV panels increased the mission range by 25%. However, if the mission follows the best path defined by the SEMS at a lower speed between 0.5 m/s and 1.0 m/s the USV range would further increase and also present the possibility of operating only with the PVs. This would mean that during daytime the USV range would be unlimited.

**Fig. 26:**
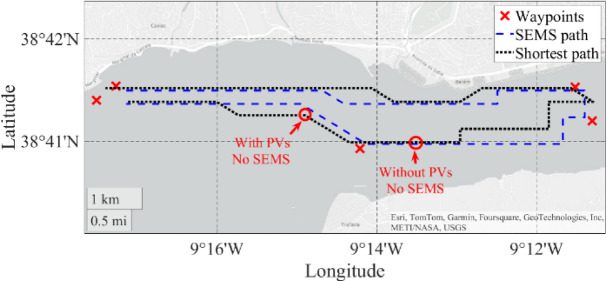
Comparison between the optimized SEMS path and the shortest path.

To evaluate the importance of the SEMS on the extension of the USV range, a simulation of the mission was carried out in parallel using a shortest path algorithm. For the same experimental environmental conditions, simulation results show that a higher consumption would be if a shortest path was selected, instead of the optimized energy path obtained using the SEMS. Figure 26 shows the comparison between the optimized SEMS path and the shortest path. Even with a small deviation between paths, the energy consumption would increase 16.4% from 2.31 to 2.69 kWh. Of course, this difference would differ if the mission was started at another local time of the day. For example, this difference would be higher if the mission had started when the water currents were opposite to the USV path.

Therefore, considering a safety margin of 20% on the batteries, the mission would stop at different points, considering or not the inclusion of PVs. These points are marked with red circles in Fig. 26 (“with PVs, no SEMS” and “without PVs, no SEMS”).

## Conclusions

This paper proposes a methodology to extend the range of marine unmanned surface vehicles (USV) for border surveillance missions. The main objectives were (a) to demonstrate the feasibility of a fossil-free low-weight catamaran-type Unmanned Marine Surface Vehicle (USV) with electric propulsion, capable of extended autonomy, (b) to develop a smart energy management system (SEMS) to govern efficiently the USV based on the mission profile and the predicted and real weather/sea conditions, and (c) to demonstrate the integrated USV and SEMS prototypes in real river and sea environments.

Two SEMS algorithms were proposed and compared. The first considers a graph approach, however, it can only handle static weather and sea conditions. The second is a new A-star algorithm with probabilistic behaviour to handle time-varying weather and sea conditions. It was verified that the A-star algorithm with probabilistic behaviour is capable of finding optimized paths with lower energy consumption than the graph-based algorithms. Depending on the starting time of the mission, it can reduce by almost half the energy consumption for the same mission.

Experimental tests conducted on the Tejo River in Lisbon, Portugal, confirmed the performance of SEMS, achieving a final energy deviation of less than 1.5% between the real and predicted values. The inclusion of the PV panels increased the mission range from 25 to 50%. However, if the mission follows the optimized path defined by the SEMS, at a lower speed between 0.5 m/s and 1.0 m/s, the USV range could be further extended, with the added possibility of operating solely with the photovoltaic panels. The proposed path defined by the developed smart energy management system led to an energy consumption decrease of about 18%, when compared with the shortest path, however, this decrease could be up to around 40% if another starting mission time was considered.

## Electronic supplementary material

Below is the link to the electronic supplementary material.

Supplementary Material 1

Supplementary Material 2

## Data Availability

Data is provided within the manuscript and data sets generated during the current study are available from the corresponding author on reasonable request.
