# Supplementary material for: A smart energy management system for surface unmanned vehicles for border surveillance missions
Source: Sci Rep. 2025 Jul 3;15:23684. doi: 10.1038/s41598-025-08579-x (PMC12222943; doi:10.1038/s41598-025-08579-x)
Supplement: Supplementary file 2 — Supplementary Material 2 [file 41598_2025_8579_MOESM2_ESM.docx]

File: SEMS4USV - Smart Energy Management System For USV.mp4

Video Legend: This video shows the experimental tests carried out to validate the developed smart energy management system and the overall hardware and software applications for the USV.
